# l-Serine Biosensor-Controlled Fermentative Production of l-Tryptophan Derivatives by *Corynebacterium glutamicum*

**DOI:** 10.3390/biology11050744

**Published:** 2022-05-13

**Authors:** Lenny Ferrer, Mahmoud Elsaraf, Melanie Mindt, Volker F. Wendisch

**Affiliations:** 1 Genetics of Prokaryotes, Faculty of Biology & CeBiTec, Bielefeld University, 33615 Bielefeld, Germany; lenny.ferrer@uni-bielefeld.de (L.F.); elsaraf.mahmoud@gmail.com (M.E.); 2 Business Unit Bioscience, Wageningen Plant Research, Wageningen University & Research, 6708 PB Wageningen, The Netherlands; melanie.mindt@wur.nl

**Keywords:** *Corynebacterium glutamicum*, metabolic engineering, biosensor, tryptophan, hydroxytryptophan, bromotryptophan, fluorotryptophan, indoles, TrpB

## Abstract

**Simple Summary:**

l-tryptophan is an amino acid found in proteins. Its derivatives, such as hydroxylated or halogenated l-tryptophans, find applications in the chemical and pharmaceutical industries, for example, in therapeutic peptides. Biotechnology provides a sustainable way for the production of l-tryptophan and its derivatives. In the final reaction of l-tryptophan biosynthesis in bacteria, such as *Corynebacterium glutamicum*, another amino acid, l-serine, is incorporated. Here, we show that *C. glutamicum* TrpB is able to convert indole derivatives, which were added to cells synthesizing l-serine, to the corresponding l-tryptophan derivatives. The gene *trpB* was expressed under the control of the l-serine-responsive transcriptional activator SerR in the *C. glutamicum* cells engineered for this fermentation process.

**Abstract:**

l-Tryptophan derivatives, such as hydroxylated or halogenated l-tryptophans, are used in therapeutic peptides and agrochemicals and as precursors of bioactive compounds, such as serotonin. l-Tryptophan biosynthesis depends on another proteinogenic amino acid, l-serine, which is condensed with indole-3-glycerophosphate by tryptophan synthase. This enzyme is composed of the α-subunit TrpA, which catalyzes the retro-aldol cleavage of indole-3-glycerol phosphate, yielding glyceraldehyde-3-phosphate and indole, and the β-subunit TrpB that catalyzes the β-substitution reaction between indole and l-serine to water and l-tryptophan. TrpA is reported as an allosteric actuator, and its absence severely attenuates TrpB activity. In this study, however, we showed that *Corynebacterium glutamicum* TrpB is catalytically active in the absence of TrpA. Overexpression of *C. glutamicum* *trpB* in a *trpBA* double deletion mutant supported growth in minimal medium only when exogenously added indole was taken up into the cell and condensed with intracellularly synthesized l-serine. The fluorescence reporter gene of an l-serine biosensor, which was based on the endogenous transcriptional activator SerR and its target promoter P*_serE_*, was replaced by *trpB*. This allowed for l-serine-dependent expression of *trpB* in an l-serine-producing strain lacking TrpA. Upon feeding of the respective indole derivatives, this strain produced the l-tryptophan derivatives 5-hydroxytryptophan, 7-bromotryptophan, and 5-fluorotryptophan.

## 1. Introduction

Amino acids and their derivatives can be produced by chemical synthesis, enzyme catalysis, or de novo by fermentation [1]. Fermentative production of the food and feed amino acids l-glutamate and l-lysine is a multi-billion USD market. The annual production of the amino acids l-tryptophan and l-serine amounts to 41,000 and 350 tons, respectively [2]. In nature, l-tryptophan is synthesized from l-serine, and structural analyses of aminoacyl-tRNA synthetases indicate that the fixation of l-tryptophan (and l-tyrosine) in the genetic code occurred late [3]. Quantum chemical calculations and biochemical experiments revealed that the enhanced redox reactivity of l-tryptophan (and l-tyrosine) protect oxidatively stressed cells from destruction by oxygen free radicals, and such a property may be shared by derivatives of l-tryptophan (such as *N*-Acetyl-l-tryptophan-*O*-ethylester) [3]. Free indole and l-tryptophan are versatile nucleophiles in chemical syntheses that can be functionalized at all non-bridgehead positions [4]. In bacteria, fungi, and plants, thousands of indole alkaloids are synthesized from l-tryptophan as secondary metabolites. Due to their chemical reactivity and physiological properties, l-tryptophan and its derivatives are sought-after compounds. Derivatives of l-tryptophan are of industrial interest because they serve as biosynthetic precursors to compounds that possess diverse biological activities, such as anticancer, antibiotic, immunosuppressant, antifungal, and phytotoxic properties, but their chemical synthesis remains to be challenging. Indole, for example, a flavor enhancer and fragrance molecule derived from l-tryptophan, has been produced by *C. glutamicum* in biotransformation and de novo fermentation processes [5,6].

Biosynthesis of amino acids is regulated according to availability and demand. In *C. glutamicum*, a number of transcriptional regulators controlling the transcription of genes and operons for amino acid biosynthesis and export are known, e.g., ArgR [7], LysG [8], ShiR [9], GlyR [10], and SerR [11,12]. Biosynthesis of l-tryptophan and the other aromatic amino acids l-tyrosine and l-phenylalanine is initiated by chorismate biosynthesis from erythrose 4-phosphate and phosphoenolpyruvate in the shikimate pathway. The enzymes encoded in the *C. glutamicum trpEGDCFBA* operon convert chorismate to l-tryptophan. In the last reaction, tryptophan synthase condenses indole-3-glycerophosphate with l-serine. This enzyme is composed of two subunits. The α-subunit TrpA catalyzes the retro-aldol cleavage of indole-3-glycerol phosphate (IGP), yielding glyceraldehyde-3-phosphate (G3P) and indole. The β-subunit TrpB yields water and l-tryptophan in a β-substitution reaction between indole and l-serine. Thus, the biosynthesis of l-serine has to be fine-tuned to match the demand not only for protein biosynthesis but also for l-tryptophan biosynthesis.

Overproduction of l-serine, an amino acid of significant biotechnological relevance owing to its expanding significance in food and the pharmaceutical and cosmetics industries [13], has been achieved by the combination of re-routing the carbon flux from the glycolytic metabolite, 3-phospho-glycerate, to the l-serine biosynthetic pathway and by the deletion of *glyR* in *C. glutamicum* [14]. GlyR activates the transcription of *glyA*-encoding l-serine hydroxymethyltransferase (SHMT) in the stationary growth phase [10]. SHMT occupies a central position in one-carbon metabolism in *C. glutamicum* by catalyzing the reversible interconversion of l-serine and tetrahydrofolate to glycine and methylenetetrahydrofolate. The activated one-carbon units generated from this enzymatic reaction are required for various cellular processes, e.g., synthesis of formylated methionine bound to the initiator tRNA^fMet^ [10]. The cleavage of l-serine to glycine by SHMT serves as the main l-serine degradation pathway in *C. glutamicum*, but due to its role in cellular physiology, the SHMT reaction cannot simply be bypassed by the addition of external metabolite. Therefore, the deletion of *glyA* in *C. glutamicum* for l-serine production has been shown to be futile and lethal to host cells [15]. This was overcome by a reduced folate supply [16] or by the deletion of *glyR* [14].

l-serine is exported from the *C. glutamicum* cell by the export protein SerE, which is encoded upstream of *serR* and transcribed in the divergent direction [12]. The transcriptional regulator protein SerR activates the transcription of *serE* when l-serine is abundant [11]. Overexpression of both *serE* and *serR* in an l-serine-base-producing strain was shown to be beneficial for l-serine production [12]. SerR is one of the transcriptional regulators used as genetically encoded biosensors for application in the metabolic engineering of *C. glutamicum*: Lrp-based biosensor for branched-chain amino acids and l-methionine [17,18,19], LysG-based biosensor for l-Lysine [20,21], ShiR-based biosensor for shikimic acid [22], GlxR-based cAMP biosensor [23], and SerR-based biosensor for l-serine [24]. The l-serine biosensor plasmid pSenSer is based on SerR and its target promoter, P*_serE_*, which is fused to a promoterless yellow fluorescent protein (EYFP) reporter gene. Binder et al., 2012 showed that pSenSer is a useful tool for visualizing intracellular l-serine concentrations [24]. Zhang et al., 2018 employed this biosensor’s fluorescence output to isolate l-serine-overproducing *C. glutamicum* cells after atmospheric and room temperature plasma mutagenesis [11].

An l-tryptophan-responsive biosensor has not yet been developed for *C. glutamicum*. In *C. glutamicum*, the transcriptional repressor LtbR binds to a 12-bp motif between the −10 promoter region and the ribosome binding site of the *trpL* gene and prevents transcription of the *trp* operon [25]. Albeit lacking a TrpR homolog, *C. glutamicum* has a putative operator sequence with similarity to the operator DNA binding sequence of the TrpR repressor of *E. coli* and was detected upstream of the −10 promoter region of the *trp* operon [26,27]. Transcription of the *trp* operon is controlled by attenuation and deletion of the leader peptide gene *trpL* and improved overproduction of l-tryptophan [28] and aromatic aldehydes [29]. Several l-tryptophan-producing *C. glutamicum* strains have been developed [30,31,32]. Typically, genes of the common chorismate pathway and the dedicated l-tryptophan branch are overexpressed, and key enzymes are feedback-desensitized variants, combined with an improved supply of precursors from the central carbon metabolism and with enhanced redox cofactor regeneration.

In this study, the production of l-tryptophan and its derivatives was based only on the reaction catalyzed by tryptophan synthase β-subunit TrpB. The β-reaction has already been employed for the preparation of a wide range of l-tryptophan derivatives [33,34,35]. As a single-catalyst platform, l-tryptophan analogue conversion from the widely available indole analogues is accessible [36,37,38]. Here, it was found that the catalytic activity of *C. glutamicum* TrpB is not attenuated in the absence of its allosteric actuator TrpA and readily reacts with various indoles and l-serine. Since l-serine is a co-substrate of the TrpB reaction and its availability has to be on par with the demand for l-tryptophan biosynthesis, a *C. glutamicum* l-serine-overproducing strain [16,39] was endowed with the l-serine biosensor to drive the expression of *trpB*. This enabled the production of l-tryptophan derivatives from intracellularly synthesized l-serine and exogenously added indole derivatives.

## 2. Materials and Methods

### 2.1. Strains and Culture Conditions

The genotypes of the bacterial strains used in this study are listed in Table 1. *Escherichia coli* DH5α [40] was used as a cloning host and for plasmid propagation. *C. glutamicum* ATCC 13032 was used as a host organism for evaluation of l-serine-responsive biosensors and production of l-tryptophan derivatives. Luria–Bertani medium was used as the standard medium for *E. coli*, brain heart infusion (BHI) medium (Difco) as the complex medium, and CGXII [41] as minimal medium for *C. glutamicum*, with 40 g L^−1^ glucose as carbon source. Whenever appropriate, the following were supplemented in the medium: kanamycin (25 µg mL^−1^), tetracycline (5 µg mL^−1^), and 1 mM IPTG. *E. coli* and *C. glutamicum* were grown at 37 °C and 30 °C, respectively, in baffled shake flasks on a rotary shaker (160 rpm or 120 rpm). For *C. glutamicum* growth experiments, overnight pre-cultures were prepared as described above, harvested, and washed with TN buffer pH 6.3 (50 mM Tris-HCl, 50 mM NaCl), and the main medium is inoculated to an optical density at 600 nm (OD_600_) of 1. Growth and production experiments with the *C. glutamicum* SER1 and SER2 were grown in CGXII minimal medium under folate-starvation condition as seed cultures and the main culture in CGXII minimal medium supplemented with 0.1 mM folate [16]. Growth medium for SER2 strain was supplemented with 0.25 g L^−1^ (1.22 mM) l-tryptophan for growth. Growth of *C. glutamicum* cultivated in 500 mL baffled shake flasks was monitored by measuring OD_600_ using a V-1200 spectrophotometer (VWR, Radnor, PA, USA). HT cultivations were performed in 48-well Flowerplates with the BioLector cultivation system (m2p-labs GmbH, Baesweiler, Germany) [42,43]. Formation of biomass was recorded as a backscatter light intensity at 620 nm wavelength, while the fluorescence of the yellow fluorescent protein mVenus NB was measured with excitation at 488 nm and emission at 520 nm.

### 2.2. Molecular Genetic Techniques, Plasmids, and Strain Construction

Standard molecular genetic techniques were performed as described elsewhere [46]. Chromosomal deletions were performed using the non-replicable suicide vector pK19*mobsacB* [47] in a two-step homologous recombination [41]. The genomic regions flanking the respective gene for homologous recombination and, unless otherwise stated, the genes for cloning were amplified from *C. glutamicum* ATCC13032 gDNA using ALLin HiFi DNA Polymerase according to manufacturer (highQu GmbH, Kraichtal, Germany). Cloning of the amplified PCR products was performed using restriction enzyme digestion and Gibson assembly [48]. The oligonucleotides and plasmids used in this study are listed in Table 2 and Table 3, respectively.

L-serine-responsive biosensors were assembled in pGP21 plasmid; pGP21 was designed based on the vector pGP2 [49]. The plasmid pGP2 was modified to contain the T7te and *rrnB* T1 terminator for the divergent expression of the biosensor parts (i.e., transcription factor and reporter gene/biosynthetic *trpB* gene). To this end, pGP21-F and pGP21-R primers (Table 2) were used to amplify the T7te and *rrnB* T1 terminator from the pRG-Duet2 [50] vector. The regulatory unit, *serR* (NCgl0581), and the intergenic region to *serE* were amplified using the primers serR-F and serE-R, while primer set mVen-F and mVen-R were used to amplify the reporter gene *mVenus NB* from pEB1-*mVenus NB* plasmid (Addgene plasmid # 103986). These DNA fragments were cloned into *Bam*HI-digested pGP21.

**Table 3:** Plasmids used in this study.

Plasmids	Description	Source
pGP2	pVWEx1 [51]-derived *E. coli*-*C. glutamicum* expression vector without *lacI^q^* and *tac* promoter; Km^R^, pHM1519 *oriV_Cg_*	[49]
pGP21	pGP2-derived *E. coli*-*C. glutamicum* shuttle vector	This work
pSenSer1	pGP21 containing *serR*, intergenic region of *serR* and *serE*, and transcriptional fusion of *serE* with *mVenus*	This work
pSenSer1-*serE*’-26 aa	pSenSer1 with single-point mutation in the *serE-mVenus transcriptional* fusion to yield a 78-bp *serE*	This work
pSenSer2-*serE*’-26 aa	pSenSer1-*serE*’-26 aa with start codon exchange of *serR* from GTG to ATG	This work
pSdS-*trpB*	pSenSer1 with *mVenus* replaced by *trpB*	This work
pK19*mobsacB*	Km^R^, *oriV_Ec_*, *sacB*, *lacZα*; *E. coli*-*C. glutamicum* shuttle vector for construction of insertion and deletion mutants in *C. glutamicum*	[47]
pK19*mobsacB* Δ*serA*	pK19*mobsacB* with a construct for in-frame deletion of *serA*	This work
pK19*mobsacB* Δ*trpBA*	pK19*mobsacB* with a construct for in-frame deletion of *trpBA*	This work
pEKEx3	Spec^R^, P*_tac_ lacI^q^*, pBL1 *oriV_Cg_*, *sacB*, *lacZα*; *E. coli*-*C. glutamicum* expression vector	[52]
pEKEx3-*trpBA*	pEKEx3 overexpressing *trpBA*	This work
pEKEx3-*trpB*	pEKEx3 overexpressing *trpB*	This work

To introduce single-point mutation into pSenSer1, site-directed mutagenesis (SDM) was performed and primers were designed according to Ref. [53]. A PCR reaction of 50 µL was prepared to contain 3 units (*PfuTurbo* DNA polymerase (Agilent Technologies, Santa Clara, CA, USA), 10 ng plasmid, 125 ng forward and reverse primers, 200 µM dNTPs, and *Pfu* DNA polymerase reaction buffer). To construct pSenSer1-*serE*’-26 aa and pSenSer2-*serE*’-26, primer sets SDM1-F/SDM1-R and SDM2-F/SDM2-R were used, respectively. To construct pSdS-*trpB*, pSenSer1 biosensor plasmid was double-digested with EcoRV and BamHI to remove *mVenus*. The *trpB* gene was then amplified with primers trpB-F and trpB-R and subsequently ligated to the linearized pSenSer1 by Gibson Assembly.

### 2.3. Quantification of l-Serine and Aromatic Compounds

Extracellular l-serine was quantified by high-performance liquid chromatography (HPLC) (1200 series, Agilent Technologies Deutschland GmbH, Böblingen, Germany). The culture supernatants were collected at specific time points and centrifuged (20,200× *g*, 10 min 4 °C) for analytical analysis. Samples for l-serine analysis were derivatized with ortho-phthaldialdehyde (OPA) (Schneider, Niermann, and Wendisch 2011). The amino acid separation was performed by a pre-column (LiChrospher 100 RP18 EC-5 µ (40 mm × 4 mm), CS-Chromatographie Service GmbH, Langerwehe, Germany) and a column (LiChrospher 100 RP18 EC-5 µ (125 mm × 4 mm), CS-Chromatographie Service GmbH, Langerwehe, Germany). Detection was carried out with a fluorescence detector (FLD G1321 A, 1200 series, Agilent Technologies) with the excitation and emission wavelengths of 230 nm and 450 nm, respectively. Aromatic compounds were analyzed from the culture supernatants according to Ref. [54]. A mobile phase gradient of buffer A (0.1% trifluoroacetic acid dissolved in water) and buffer B (acetonitrile) was used, and detection was performed with Diode Array Detector (DAD G1315B, 1200 series, Agilent Technologies) with scanning window of 210 nm to 330 nm.

For the quantification of intracellular l-serine, 1 mL of broth for each strain grown in minimal medium was collected at indicated time points. It was then centrifuged for 10 min at 20,200× *g*, 4 °C. Pellets were treated with 5% HClO_4_ in an ice bath for 30 min. After centrifuging at 20,200× *g* for 10 min and 4 °C, the supernatant was neutralized with K_2_CO_3_ solution and centrifuged again at 13,000 rpm for 10 min, 4 °C. The supernatant was then ready for amino acid quantification or to be stored at −20 °C [55]. The biomass concentration was determined according to the correlation CDW = 0.353 OD [56].

Identification of 5-fluorotryptophan (5-FTP) was performed by LC–MS analysis. Culture supernatants of strain SER2 pSdS-*trpB* cultivated in presence of 5-fluoroindole (5-FI) were diluted with methanol and centrifuged as described above. Five microliters of clear supernatants or a 5-FI standard (TCI Chemicals, Germany GmbH; monoisotopic mass: 135.05) were analyzed using the LC-PDA-LTQ-Orbitrap FTMS system (ThermoScientific), equipped with Acquity UPLC (H-class), Acquity elambda photodiode array detector (220–600 nm), and an LTQ/Orbitrap XL hybrid mass spectrometer equipped with an electrospray ionizer (ESI). FTMS full scans (*m/z* 90.00–1350.00) were recorded with a resolution of 60,000. Separation was carried out on a reversed-phase column (Luna C18/2, 3μ, 2.0 × 150 mm; Phenomenex, Torrance, CA, USA) at 40 °C using eluent A (ultrapure water: formic acid (1000:1, *v*/*v*)) and eluent B (acetonitrile: formic acid (1000:1, *v*/*v*)) at a flow rate of 0.19 mL/min. A linear gradient from 5 to 75% B in 45 min was applied, followed by 15 min washing and equilibration.

## 3. Results

### 3.1. Design and Construction of an l-Serine-Responsive Biosensor

**Figure 1:**
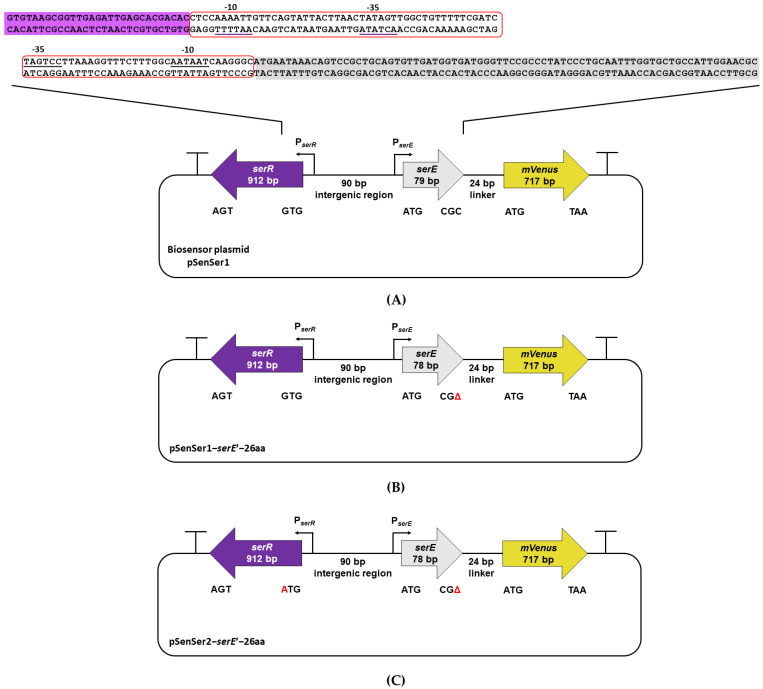
Schematic representation of l-serine biosensor components. (**A**) The transcriptional activator SerR (gene in violet) is expressed from its native promoter (P*_serR_*). SerR activates the expression of the promoter of *serE* (in gray) at elevated intracellular l-serine concentrations. The intergenic region between *serR* and *serE* (boxed in red) and the putative −10 and −35 regulatory sequence are labeled and underlined. (**B**) pSenSer1-*serE*’-26aa with a nucleotide deletion leading to shortened SerE peptide and (**C**) pSenSer2-*serE*’-26aa replacing the translational start codon of *serR* from GTG to the canonical ATG.

To design a biosensor that responds to elevated intracellular l-serine concentrations, the *C. glutamicum* native SerR-P*_serE_* biosensor first described and used by Binder et al., 2012 as a tool for the visualization of intracellular l-serine [24] was adapted. The EYFP reporter gene was replaced with the *mVenus* gene since *mVenus* has been reported to be a rapidly maturing monomer with moderate acid sensitivity as compared to EYFP [57]. The resulting biosensor plasmid called pSenSer1 (Figure 1) was transformed into the SER1 strain, a strain secreting l-serine under the folate-starvation condition [16], to evaluate the capability of the biosensor in monitoring the intracellular accumulation of its effector molecule.

**Figure 2:**
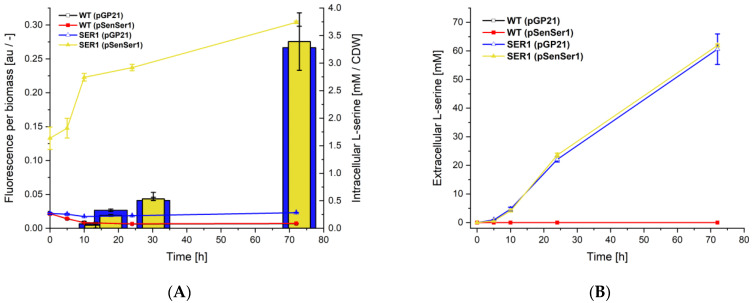
Characterization of l-serine biosensor plasmid pSenSer1 in the *C. glutamicum* ATCC 13032 (WT; square) and the recombinant l-serine producer strain SER1 (triangle). WT and SER1 were transformed with either empty vector pGP21 (empty symbols) or pSenSer1 (filled symbols). The seed cultures were first grown in CGXII minimal medium without folate to induce folate starvation before cultivating in the main culture using CGXII minimal medium supplemented with 0.1 mM folate. (**A**) The y-axis on the left shows mVenus fluorescence signal normalized to the biomass (backscatter measured in the BioLector; lines). The y-axis on the right shows intracellular l-serine concentrations (bars) determined at 5 h, 10 h, 24 h, and 72 h. (**B**) Extracellular l-serine concentrations were measured at cultivation time points when intracellular concentrations were measured. Means and errors of three cultures are presented.

Strains *C. glutamicum* WT and SER1 were transformed with either the empty vector pGP21 or biosensor plasmid pSenSer1 and were grown in CGXII minimal medium supplemented with 0.1 mM folate in the BioLector system to monitor fluorescence, growth, and l-serine production. To determine the correlation between the fluorescence output signal and effector concentration, intracellular l-serine concentrations at certain time points were measured (Figure 2A). As expected, no discernable fluorescence signal was observed using WT strains and SER1 (pGP21), while SER1 harboring pSenSer1 exhibited fluorescence (Figure 2A). Measurable amounts of l-serine were detected in the cytosol of both SER1 (pGP21) and SER1 (pSenSer1), while l-serine was not detected in the WT strains (at concentration ˂ 0.12 mM). Interestingly, until 5 h of cultivation, only autofluorescence was detected in the SER1 (pSenSer1) strain and only at 10 h and later, a significant fluorescence was recorded. This may indicate that it takes time for enough l-serine to induce *mVenus* gene expression and, subsequently, to accumulate mVenus fluorescence reporter protein. The highest fluorescence was recorded at 72 h, which also corresponded to the highest intracellular l-serine measured. Similarly, Stolz et al. also observed a graded rise in l-serine production over time when the SER1 strain was cultivated under the folate-limiting condition in shake flasks [16]. There was no significant difference in the production concentration of the two SER1 strains (i.e., SER1 empty vector strain and the sensor strain produced 60.6 ± 5.3 and 61.9 ± 0.5 mM l-serine, respectively, in BioLector cultivation), suggesting that l-serine production is not affected by the biosensor plasmid (Figure 2B).

**Figure 3:**
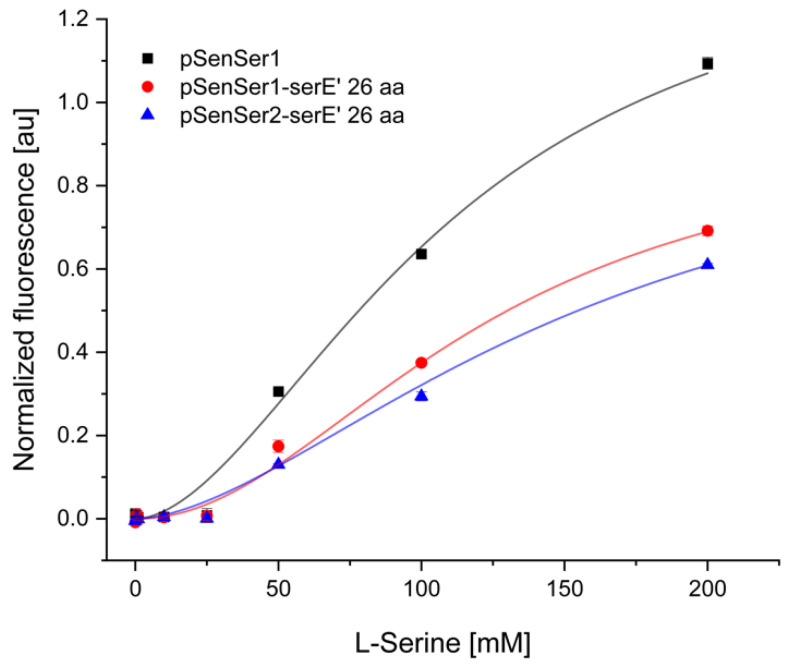
Dose–response curve of *C. glutamicum* ATCC 13032 Δ*serA* with different l-serine-responsive biosensor plasmids in cultivations with different concentrations of extracellularly added l-serine. The biosensor fluorescence output at each effector concentration was determined after subtraction of the cell autofluorescence and by normalization to the cell density. The curves were fitted to the Hill equation. Means and error bars were determined from the analysis of three independent cultures.

Since the applicability of a biosensor requires that the relationship between the effector molecule and the reporter output is defined, the dose–response of pSenSer1 was analyzed in an l-serine auxotrophic mutant that was constructed by the deletion of the native *serA* gene in the wild-type *C. glutamicum*. Strain *C. glutamicum* ATCC 13032 Δ*serA* was, therefore, unable to synthesize l-serine; thus, the exogenous addition of l-serine to the medium could be used to modulate the intracellular l-serine concentration detectable by pSenSer1. As shown in Figure 3, a clear increase in pSenSer1 output was measured with increasing concentrations of l-serine supplemented. A plateau was not reached with the highest l-serine concentration tested, i.e., 200 mM.

Two variants of the pSenSer1 biosensor were constructed and tested. Plasmid pSenSer1 contains a transcriptional fusion of the promoter of *serE* with the promoterless *mVenus* gene. The construct allows for transcription of an mRNA comprised of 79 base pairs (bp) of *serE*, a linker sequence of 24 bp, and 717 bp of the *mVenus* coding sequence [24]. Translation of this mRNA may, thus, not only lead to a full-length mVenus fluorescence reporter protein but, in addition, also to a peptide initiating at the translational start codon of *serE*. To test if the putative synthesis of this peptide extends well into the *mVenus* gene, but in a different open reading frame, the last nucleotide of the 79 bp *serE* sequence was deleted in pSenSer1-*serE*’-26aa. By this manner, the resulting mRNA only codes for a defined short SerE peptide of 26 amino acids in addition to the full-length mVenus protein (Figure 1B). In the second variant, pSenSer2-*serE*’-26aa (Figure 1C), the translational start codon of the regulatory gene *serR* was changed from the less-preferred GTG to the canonical ATG. This change was expected to increase the SerR regulator protein concentration, which may positively affect the biosensor amplitude.

The variants pSenSer1-*serE*’-26aa and pSenSer2-*serE*’-26aa were used to transform the l-serine auxotrophic strain *C. glutamicum* ATCC 13032 Δ*serA* and used to compare with ATCC 13032 Δ*serA* (pSenSer1). A sigmoidal dose–response curve was observed for all three strains (Figure 3). The finding of little fluorescence when 0.5 to 25 mM of l-serine was supplemented may be due to co-metabolization of l-serine with glucose at high utilization rates, as observed previously [58]. The biosensor output was highest with the highest tested concentration of l-serine (200 mM). Comparing the three biosensor variants revealed that the signal amplitude was highest with pSenSer1, followed by pSenSer1-*serE*’-26aa before pSenSer2-*serE*’-26aa. Thus, while all three biosensors were suitable, pSenSer1 was chosen for experiments aiming at dynamic expression regulation in response to intracellular l-serine.

### 3.2. Construction of an Indole-Essential C. glutamicum Strain

**Scheme 1:**
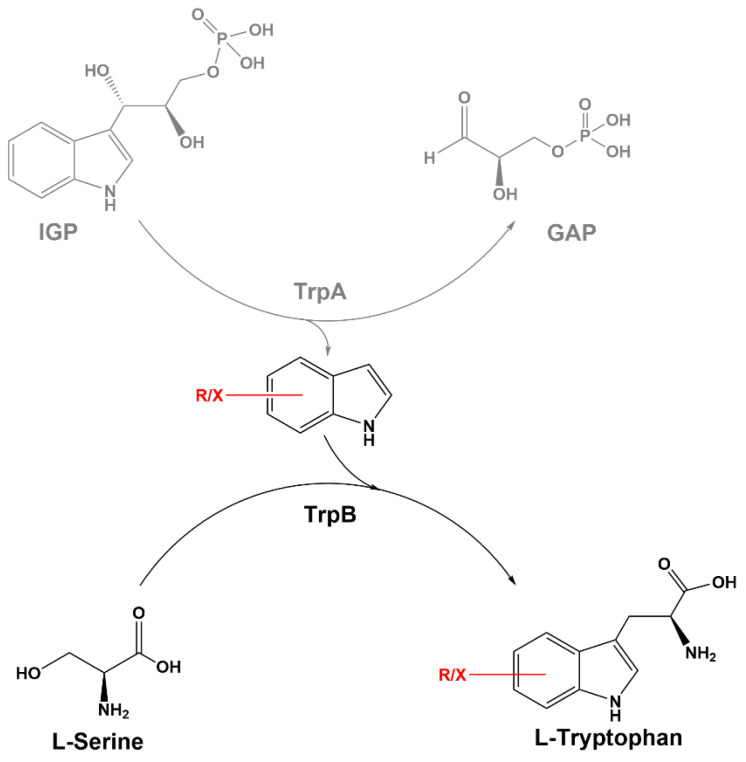
Tryptophan synthase enzymatic reaction. TrpA (top, in gray) catalyzes the retro-aldol cleavage on IGP (indole-3-glycerol phosphate) to G3P (glyceraldehyde-3-phosphate). Indole diffuses to the TrpB subunit (bottom, in black), which performs the β-substitution reaction between indole and l-serine, releasing water (not shown) and l-tryptophan. Decorations in the indole moiety are shown in red.

l-Serine is condensed with IGP to yield l-tryptophan in the ultimate reaction of l-tryptophan biosynthesis. More specifically, tryptophan synthase subunit A (TrpA) cleaves IGP to yield G3P and indole, and wherein the latter is condensed with l-serine in a β-substitution reaction, releasing water by subunit B (TrpB) (Scheme 1).

**Figure 4:**
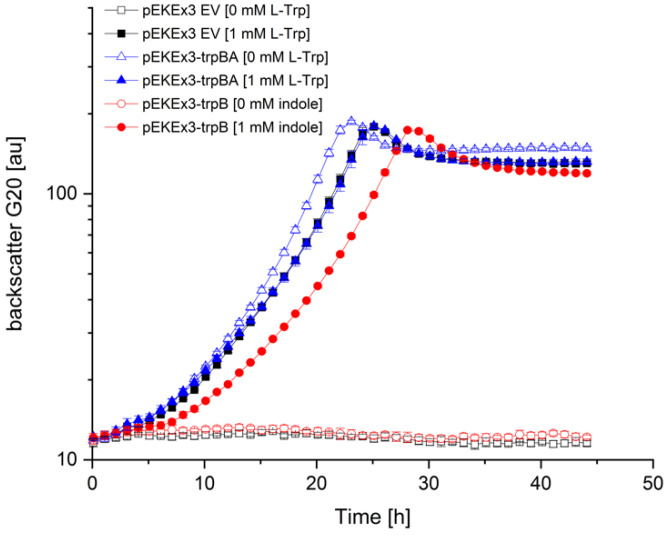
Growth of *C. glutamicum* C1* Δ*trpBA* deletion mutant strain in standard CGXII minimal medium with 40 g L^−1^ glucose. Empty vector pEKEx3 (black square), pEKEx3-*trpBA* (blue triangle), or pEKEx3-*trpB* (red circle) was transformed to C1* Δ*trpBA*. Effect of either l-tryptophan (l-Trp) or indole supplementation (filled symbols) in the growth of the constructed strains was evaluated in a BioLector system. Data presented are means and standard deviations of three independent cultivations.

While extensive studies on the mechanism of the enzymatic reaction of tryptophan synthase have shown that the two subunits of the complex have low catalytic efficiencies outside the complex [59], it was tested if the absence of TrpA can be compensated for by extracellular addition of indole. To this end, the genes for both subunits *trpBA* were deleted in a base *C. glutamicum* C1* strain, yielding an l-tryptophan auxotrophic strain. C1* was used as a base strain to avoid possible effects of l-serine overproduction in strain SER2. This strain was transformed with the empty IPTG-inducible expression plasmid pEKEx3 (negative control), pEKEx3-*trpBA* (positive control of genetic complementation), and with pEKEx3-*trpB*. As expected, all strains grew when supplemented with 1 mM l-tryptophan, whereas only the genetic complementation strain *C. glutamicum* C1* Δ*trpBA* (pEKEx3-*trpBA*) could grow without l-tryptophan supplementation (Figure 4). Notably, strain C1* Δ*trpBA* (pEKEx3-*trpB*), which lacks *trpA*, could grow when induced with 1 mM IPTG and supplemented with 1 mM indole. This indole-essential growth showed that catalysis by *C. glutamicum* TrpB—even outside of the native tryptophan synthase complex with TrpA—is sufficient to provide l-tryptophan for growth.

### 3.3. On-Demand Conversion of Indole and l-Serine to l-Tryptophan via l-Serine-Responsive Biosensor

**Figure 5:**
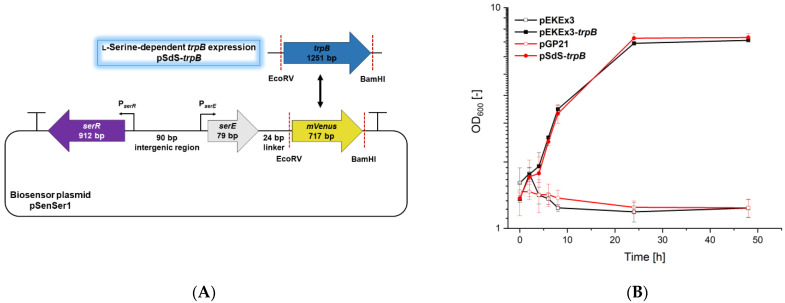
(**A**) Construction of the l-serine-switch plasmid pSdS-*trpB* for l-serine-dependent *trpB* expression. Biosensor plasmid pSenSer1 was double-digested with EcoRV and BamHI to replace the *mVenus* with *trpB* gene. (**B**) Growth of SER2 (pEKEx3-*trpB*) (black filled squares) and SER2 (pSdS-*trpB*) (red filled circles) and their corresponding control strains with empty vector in hollow symbols. Cells were grown under l-serine-producing conditions (i.e., folate starvation) in CGXII minimal medium with 1 mM indole supplementation. Values are means and errors of three cultures.

After establishing that *C. glutamicum* TrpB can salvage the growth of an l-tryptophan-auxotrophic mutant strain upon *trpB* overexpression using an IPTG-inducible plasmid and indole supplementation, *trpB* was cloned into pSenSer1 plasmid for l-serine-controlled expression, replacing *mVenus* (Figure 5A). The constructed pSdS-*trpB* was used in the following experiments to dynamically control the expression of the native *trpB* gene according to intracellular l-serine availability. In comparison, an IPTG-inducible *trpB* expression vector (pEKEx3-*trpB*) was utilized. When the switch-plasmid pSdS-*trpB* was used to transform l-serine-producing strain SER2 (Table 1), supplementation of 1 mM indole was sufficient to allow for growth, while the empty vector carrying negative control strain SER2 (pGP21) could not grow (Figure 5B). Furthermore, there was no observable difference in growth behavior between SER2 carrying the switch-plasmid pSdS-*trpB* for dynamic regulation or the fully induced IPTG-regulated plasmid pEKEx3-*trpB*. This indicated that strain SER2 provided enough l-serine and that the switch-plasmid pSdS-*trpB* was sufficient to promote l-tryptophan biosynthesis from exogenous indole and intracellular l-serine. Since both strains grew comparably (Figure 5B), we continued to use SER2 (pSdS-*trpB*) for further experiments.

### 3.4. Fermentative Production of l-Tryptophan Derivatives by C. glutamicum SER2 (pSdS-trpB) from Indole Derivatives

In principle, the tryptophan synthase β-subunit TrpB is able to react with many indole analogues [60] in the absence of TrpA (Scheme 1). After having shown that *C. glutamicum* TrpB efficiently converts indole to l-tryptophan in the absence of TrpA, it was tested if *C. glutamicum* TrpB is promiscuous with respect to the substrate indole, i.e., whether it also accepts indole analogues.

**Figure 6:**
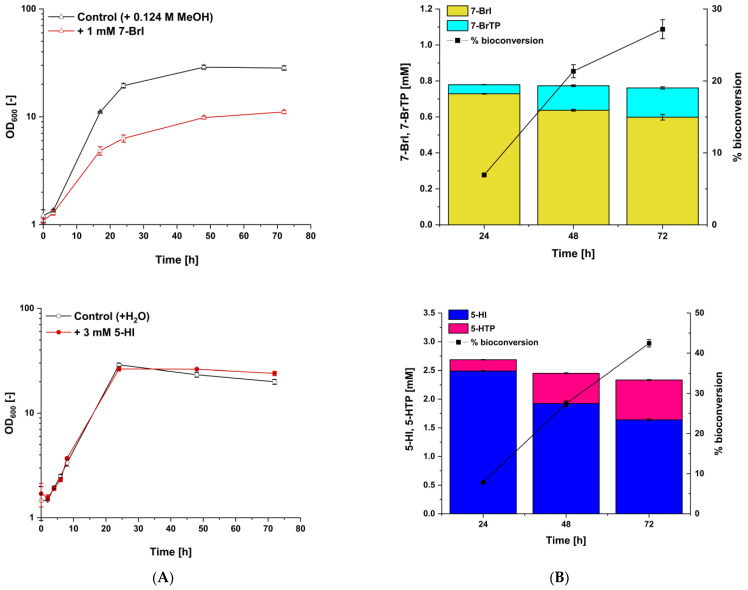
Growth (**A**) and production (**B**) of l-tryptophan derivatives in shake flask. Values and error bars represent means and standard deviations from 3 replicate cultivations with supernatants analyzed at indicated time points for HPLC. As a control, either water (solvent of 5-HI) or 0.124 M MeOH (solvent of 7-BrI) was used. Error bars indicate the standard deviations of the means of three replicates.

**Figure 7:**
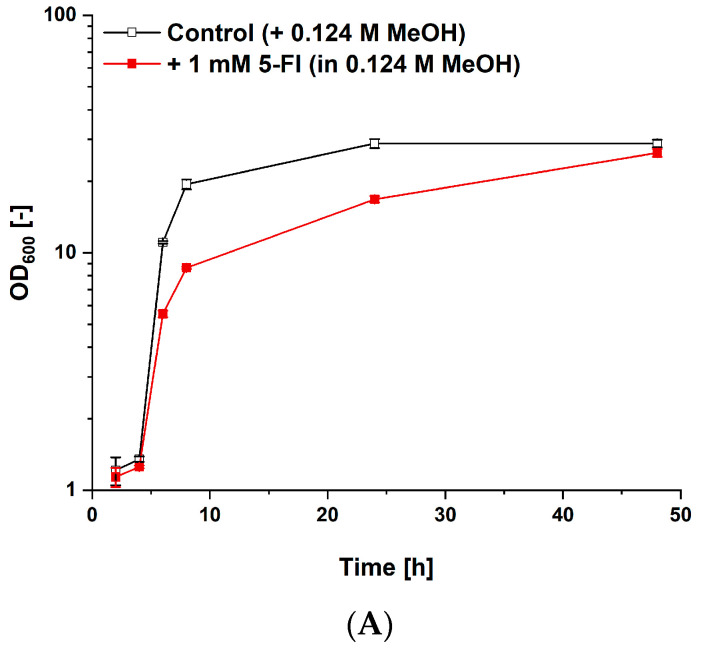

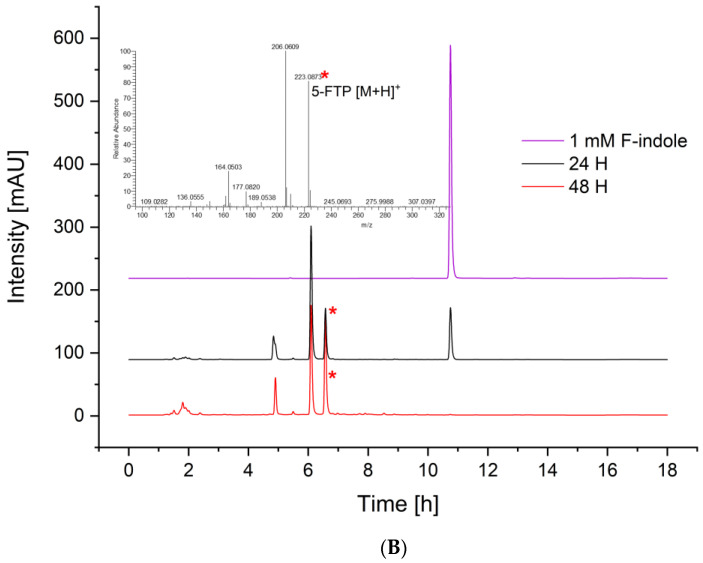
(**A**) Growth of SER2 (pSdS-*trpB*) cultivated with or without 1 mM 5-FI. Data are means and errors of three cultivations. (**B**) Conversion of 5-FI to 5-FTP by HPLC and MS analysis. The degree of conversion of 1 mM 5-FI to its corresponding 5-FTP was determined by the disappearance of 5-FI peak during HPLC analysis [retention time (RT) = 10.8 min] and appearance of the peak at RT = 6.6 min. Structural identity of the peak at RT = 6.6 min was confirmed by LC–MS analysis. Mass spectrum showed a clear peak (red asterisk) with a mass of 223.08045, which corresponds to 5-FTP [M+H]^+^. Exemplary data from three independent cultivations are given.

To this end, either 5-fluoroindole (5-FI), 7-bromoindole (7-BrI), or 5-hydroxyindole (5-HI) were added exogenously as substrates to *C. glutamicum* SER2 (pSdS-*trpB*) cells growing in minimal medium supplemented with 0.25 g L^−1^ (1.22 mM) l-tryptophan. Growth was affected by 1 mM 5-FI and 1 mM 7-BrI, but not by 3 mM 5-HI (Figure 6A and Figure 7A). Furthermore, growth was also inhibited by the product 5-HTP (Supplementary Figure S1), as was shown previously for 7-bromo-l-tryptophan (7-BrTP) [61]. In all the cultivations, about 50 to 60 mM of l-serine was produced, and the glucose was completely consumed after 48 h (data not shown). The conversion of the indole derivatives to the respective l-tryptophan derivatives 5-fluoro-l-tryptophan (5-FTP), 7-BrTP, or 5-hydroxy-l-tryptophan (5-HTP) was followed by HPLC. Quantification of 5-HI, 5-HTP, 7-BrI, 7-BrTP, and 5-FI was performed using commercially obtained standards. The formation of 5-FTP was shown by LC–MS analysis (Figure 7B).

Upon addition of 1 mM 7-BrI and 3 mM 5-HI, *C. glutamicum* SER2 (pSdS-*trpB*) produced 0.16 ± 0.01 mM 7-BrTP (27.19 ± 1.32% conversion) and 0.70 mM ± 0.01 mM 5-HTP (42 ± 0.91% conversion), respectively, after 72 h. Due to the lack of an available chemical standard for 5-FTP, the supernatants of *C. glutamicum* SER2 (pSdS-*trpB*) cultivated with 5-FI were subjected to LC–MS analysis (Figure 7; Supplementary Figure S2). Extracts revealed a mass of 223.080405, which corresponded to 5-FTP [M+H]^+^. Moreover, 5-FI (the HPLC peak at RT = 10.8 min) could not be detected from 24 h to 48 h, indicating depletion of 5-FI. Hence, the LC–MS analysis confirmed the conversion of 5-FI to 5-FTP by SER2 (pSdS-*trpB*).

Taken together, the native TrpB of *C. glutamicum* was shown to be active not only without TrpA as a stand-alone enzyme, but also to accept indole analogues as substrates in addition to its native substrate indole. On-demand expression of *trpB* gene was achieved by placing it under the control of an l-serine-responsive biosensor, and this strategy has been demonstrated to be useful in producing l-tryptophan derivatives of importance in industry, such as 7-BrTP, 5-HTP, and 5-FTP.

## 4. Discussion

The production of l-tryptophan derivatives 5-FTP, 7-BrTP, and 5-HTP was achieved by applying the l-serine-dependent expression of the native *trpB* from *C. glutamicum* in an l-serine-overproducing *C. glutamicum* strain. l-tryptophan scaffolds, l-tryptophan derivatives with decorations in their indole moiety, are widespread in bioactive natural products. The synthesis of l-tryptophan analogues such as the ones produced in this study is of interest because such functionalized derivatives lead to modified properties of biological effects that may subsequently lead to enhanced and/or novel pharmaceutically active compounds. Due to the inherent reactivity and oxidative nature of the indole ring of l-tryptophan, l-tryptophan stimulates oxidative reactions, electrophilic aromatic substitutions, and alkylation reactions [62]. However, in order to achieve l-tryptophan diversification, several different enzymes would be needed for each modification reaction, complicating the process.

Employing TrpB as a versatile catalyst to facilitate the production of l-tryptophan analogues offers a simplified route as compared to utilizing different enzymes to introduce the functional group in l-tryptophan. The unprecedented discovery that *C. glutamicum* TrpB efficiently converts indole to l-tryptophan even in the absence of its allosteric activator, TrpA, is of significance because it revealed the possibility of TrpB as being a promiscuous enzyme that accepts indole analogues. In recent years, the significance of tryptophan synthase as a biocatalytic platform has been largely investigated by the group of 2018 Nobel Prize Laureate Frances Arnold. Only the β-subunit of the tryptophan synthase catalyzes the formation of l-tryptophan derivatives. In order to release the allosteric modulation of TrpB from the α-subunit, directed evolution on TrpB was applied to identify mutations that emulate the effect of TrpA binding and to allow the high catalytic activity of TrpB in isolation. As a result, TrpB progenies were developed that have efficient stand-alone activity not only with indole as a substrate but also allowed systematic expansion to indole analogues [60]. TrpBs from thermophilic and hyperthermophilic organisms have been extensively subjected to directed evolution to recapitulate allosteric activation in the absence of TrpA. TrpB from thermophilic organisms [EC 4.2.1.122] has been described to possess indole-salvaging activity and has a lower *Km* for indoles compared to the TrpB found in mesophilic organisms [EC 4.2.1.20] (https://www.brenda-enzymes.org/enzyme.php?ecno=4.2.1.122, accessed on 4 April 2022). Besides the inherent property of thermophilic TrpB, choosing TrpB from organisms that thrive at relatively high temperatures is strategically advantageous because its thermostability simplifies enzyme purification from a mesophilic *E. coli* host and, subsequently, allows screening at elevated temperatures [63]. For instance, a TrpB mutant from *Pyrococcus furiosus Pf*TrpB^0B2^ with 83-fold improved catalytic efficiency compared to the wild-type was obtained after the introduction of mutations that were found to accelerate catalysis through the same mechanism as TrpA effector binding [63,64].

Our approach also yielded 5-FTP. Fluorination at different positions of the indole ring of l-tryptophan results in altered intrinsic properties due to the high strength and polarization of the C–F bond. Fluorinated tryptophan analogues are usually used as “intrinsic” probes of protein local structures; for instance, the absorbance spectra of 5-FTP extend to longer wavelengths than l-tryptophan itself and, hence, enable preferential excitation of the fluorinated analogue in fluorescence experiments [65]. To the best of our knowledge, no report has been made on the occurrence of a nucleophilic halogenase that specifically fluorinates l-tryptophan. There is, however, a report on a fluorinating enzyme 5′-fluoro-5′deoxyadenosine synthase from the soil bacterium *Streptomyces cattleya* [66], which participates in the biosynthesis of fluoroacetate and fluorothreonine [67]. On the other hand, brominated compounds, such as 7-BrTP, are precursors of TMC-95A, and it may be a promising candidate for tumor and inflammation therapies, because it is biologically active as a protease inhibitor against the chymotrypsin-like, trypsin-like, and peptidyl-glutamyl-peptide-hydrolyzing activities of the 20S proteasome of eukaryotic cells [68,69]. The fermentation process for 7-BrTP production based on halogenase enzymes RebH and RebF from *Lechevalieria aerocolonigenes* in a recombinant *C. glutamicum* strain has been achieved before [61].

A more simplistic and convenient approach in incorporating a halogen into l-tryptophan has been previously described by expressing the tryptophan synthase from *Salmonella enterica* on a high copy number plasmid in *E. coli*. Treating the cell lysates as synthetic reagents and mixing them with haloindole and serine in a buffer resulted in successful syntheses of 4-, 5-, 6-, or 7-halo l-tryptophan [34]. Notably, the highest yield was afforded by 5-FTP (63% after one cycle with enzyme and improved to a 100% yield after two further reaction cycles) compared to all the other analogues tested. In our process, no 5-FI was detected in the samples after 48 h, unlike in the case of 5-HI and 7-BrI (Figure 6 and Figure 7). It was, therefore, inferred that the conversion yield to 5-FTP, particularly in our biotransformation process, is high. Goss and Newill 2006 rationalized that the shorter the carbon–halogen bond, the greater the ease of conversion, as was observed in the trend 5-FI > 5-ClI > 5-BrI, and that comparatively lower yields could be postulated for 4- or 7-substituted indoles due to steric hindrance [34]. This may also be the explanation why, in this study, less than 50% biotransformation for both 5-HI and 7-BrI was observed and, although quantification was not possible for 5-FTP due to a lack of standard, the 5-FI peak was gone after 48 h. The process described in this study does not require prior cell lysis, reducing manual labor, and it decreases the cost because l-serine is already produced de novo from cheap sugar.

5-HTP is a metabolic intermediate in the biosynthesis of the neurotransmitter serotonin. It is prepared either by extraction from Ghana seeds or through the enzymatic reaction catalyzed by l-tryptophan-5-hydroxylase with l-tryptophan as the direct precursor. However, the extraction method generates pollutants to the environment because of the organic solvents used, and the T5H for biosynthesis is unstable when expressed in microorganisms and requires a special cofactor 5,6,7,8-tetrahydrobiopterin that must be generated through additional enzymatic reactions [70]. Recently, directed evolution was performed on *E. coli* tryptophan synthase to generate a mutant with increased catalytic activity towards 5-HTP conversion from l-serine and 5-HI, reaching a yield of 86.7% [71]. In this study, 42 ± 0.91% conversion was observed for 5-HTP. In the future, directed evolution may also be applied to *C. glutamicum* TrpB to improve its catalytic efficiency towards 5-HI.

As far as we are aware, this is the first study using an l-serine-responsive biosensor for an on-demand production platform for l-tryptophan derivatives through dynamic expression control of the biosynthetic gene *trpB*. Moreover, we provide a first proof of concept that *C. glutamicum* TrpB accepts not only indole but also indole analogues as substrates. Since the C–C-bond-forming reaction for the formation of l-tryptophan analogues is only catalyzed by the β-subunit of the tryptophan synthase, TrpA was bypassed, and it was shown that *C. glutamicum* TrpB is catalytically efficient as a stand-alone enzyme for the conversion of l-serine and indole (derivatives) to l-tryptophan (derivatives). Until now, it has been widely described that native TrpBs lose most of their activity in the absence of allosteric activation from their corresponding TrpAs [72,73], and both TrpA and TrpB had to be present for the activity of native, unevolved tryptophan synthase towards the synthesis of l-tryptophan derivatives. However, the likelihood that *C. glutamicum* TrpB activity can be further increased upon complexing with TrpA cannot be disregarded, and further detailed enzymatic analysis may have to be performed in the future.

In this study, the ability of the SerR-based l-serine biosensor for dynamic pathway control was harnessed in order to produce l-tryptophan analogues on-demand in an l-serine *C. glutamicum* overproducer. The specificity of the l-serine biosensor to cytosolic l-serine was shown before by Binder et al., 2012 in a peptide-dose assay wherein the addition of Ser3-OH resulted in fluorescent cultures, while this was not the case for either Ala–Ala or Thr–Ala [24]. The sensor transfer curve or the characterization of the output response of pSenSer1 and its derivatives with its cognate effector l-serine was achieved by quantifying the fluorescent output response in relation to the supplied l-serine in an l-serine-auxotrophic mutant. l-serine is known to be channeled into the central metabolism by several organisms [74]; hence, the fluorescence output could only be related to free l-serine inside the cells. Netzer et al., 2004 described that the co-utilization of glucose and l-serine in *C. glutamicum* has three distinct phases: first, cellular growth occurs during the co-metabolism of both glucose and l-serine; second, the cells enter a non-growth phase while l-serine is still being utilized; and, last, the cells stop utilizing l-serine [58]. The intracellular fate of l-serine during the second phase was determined via isotopomer labelling, and it was found that l-serine is either utilized for the synthesis of other cellular metabolites via pyruvate or oxidized to produce energy for maintenance. Similar to the described second phase that was observed in this study, the maximum OD_600_ was reached at 25 mM l-serine, but a significant signal-to-noise fluorescence ratio was only observed at 50 mM l-serine. From 50 mM to 200 mM l-serine, the biosensor showed a graded-fluorescence output with increased l-serine supplementation. In other words, *mVenus* is expressed when the cells stopped growing and l-serine is still consumed but no longer utilized for growth. This makes the system suitable for the dynamic pathway control as it allows sensing the significantly elevated intracellular l-serine level and, subsequently, causes the expression of the output gene.

The SerR transcriptional activator employed in pSenSer1 belongs to the family of LysR-type transcriptional regulators (LTTRs). LTTRs are highly conserved and ubiquitous among bacteria and described as global transcriptional regulators, acting as either activators or repressors of single genes or genes in an operon. They were also observed to be often divergently transcribed to their target gene, but could also be located elsewhere on the bacterial chromosome [75]. While the exact DNA-binding sequence of SerR upstream of *serE* is unknown, LTTR broadly binds at −40 to −20 bp as the activation site of its cognate regulated gene [75]. Nevertheless, an electrophoretic mobility shift assay (EMSA) confirmed that SerR binds to the upstream region of *serE* [12]. Knowledge on the accurate transcription factor binding sequence is useful in tuning the biosensor response [76] and may be helpful in future fine-tuning of the l-serine biosensor constructed here.

All three constructed l-serine-responsive biosensors, namely pSenSer1, pSenSer1-*serE*’-26aa, and pSenSer2-*serE*’-26aa, exhibited varied dynamic range, i.e., the difference between the minimum response value and the maximum output response in the dose–response curve, although the maximum response saturation was not reached during the l-serine feeding experiment. Therefore, each of the three could be utilized depending on the goal of the application. Sensor pSenSer2-*serE*’-26aa, for instance, could be used when a tighter response is needed. Furthermore, pSenSer1 was chosen to dynamically express *trpB* since the largest signal amplitude was observed using this biosensor. In this way, the production of l-tryptophan analogues was coupled to the elevated intracellular l-serine as sensed by the biosensor.

## 5. Patents

Patent applications regarding the results described here were not filed.

## Figures and Tables

**Table 1:** Bacterial strains used in this study.

Strain	Description	Source
*Escherichia coli*		
DH5α	Δ*lacU169* (*ØdlacZΔM15*), *supE44*, *hsdR1d7*, *recA1*, *endA1*, *gyrA96*, *thi-1*, *relA1*	[40]
S17-1	*recA pro hsdR RP4-2-*Tc::Mu-Km::Tn7	[44]
*Corynebacterium glutamicum*		
ATCC 13032	*C. glutamicum* wild-type strain	ATCC
ATCC 13032 Δ*serA*	ATCC 13032 derivative with *serA* in-frame deletion, l-serine auxotroph	This work
C1*	Genome-reduced chassis strain of ATCC 13032	[45]
C1* Δ*trpBA*	C1* derivative with *trpBA* in-frame deletion, l-tryptophan auxotroph	This work
SER1	ATCC13032 derivative with in-frame deletion of *sdaA* and *pabABC* and overexpression of *serA*(fbr), *serB*, *serC*, tetracycline-resistant, l-serine overproducer	[16]
SER2	SER1 with in-frame deletion of native *trpBA*	This work

**Table 2:** Oligonucleotides used in this study.

Name	Oligonucleotide Sequence (5′ to 3′)
pGP21-F	CAACAGAGTTTGTA**GAAACGCAAAAAGGCC**
pGP21-R	GCTCGGTACCCGGGGATCCT**AGCCCGCGGAGTACCGC**
mVen-F	GGTGCTGCCATTGGAACGCTGAACTTTAAGAAGGAGATATCAT**ATGAGTAAAGGAGAAGAACTTTTCACTGGAGTTGTCC**
mVen-R	CGCGGTACTCCGCGGGCTAGCGCGGATCCGCG**TTATTGTATAGTTCATCCA** **TGCCATGTGTAATCCCAG**
serR-F	ATTCGAGCTCGGTACCCGGGTCAC**TCTACTAGACGAGCCTCCAAATAAGAT** **CTTTG**
serE-R	AGTTCTTCTCCTTTACTCAT**ATGATATCTCCTTCTTAAAGTTCAGCGTTCCAA** **TGGCAGCACCAAATTG**
SenstrpB-F	CTGAACTTTAAGAAGGAGATATCAT**ATGACTGAAAAAGAAAACTTGGGCG** **GCTC**
SenstrpB-R	GTACTCCGCGGGCTAGCGCGGATCCGCG**TCATCGGTTGTCCTTCAGGATC** **AGTTCTG**
SDM1-F	CTCCTTCTTAAAGTTCACGTTCCAATGGCAGCAC
SDM1-R	GATATCTCCTTCTTAAAGTTCACGTTCCAATGGCAGCAC
SDM2-F	TGAACAATTTTGGAGGTGTCATGCTCAATCTCAA
SDM2-R	TTGAGATTGAGCATGACACCTCCAAAATTGTTCA
serAup-F	GCATGCCTGCAGGTCGACTCTA**GAGTACGAGACCAGACACACGTGACAAA** **AAAT**
serAup-R	GGACTCAACACGCAGGATCAGGACA**CGTCAACCCAACGGACTTCTACTGC**
serAdn-F	GCAGTAGAAGTCCGTTGGGTTGACG**TGTCCTGATCCTGCGTGTTGAGTC**
serAdn-R	AGTGAATTCGAGCTCGGTACCCGGG**TTAAGCACCGTTGTCAGACCAGTCG**
serA-conf-F	TAAAATGGCGTTAAATGGCGCGAAATGG
serA-conf-R	GGGTAAAGTGCATGAAACTCACGAGTGAG
trpBA-F	**CCTGCAGGTCGACTCTAGAG**GAAAGGAGGCCCTTCAGATGACTGAAAAAGAAAACTTGGGCGGC
trpBA-R	AAAACGACGGCCAGTGAATT**CTAAACCTTCTTGGTCGCTGCCTTCA**
trpB-F	**CCTGCAGGTCGACTCTAGAG**GAAAGGAGGCCCTTCAGATGACTGAAAAAGAAAACTTGGGCGGC
trpB-R	AAAACGACGGCCAGTGAATTTCAT**CGGTTGTCCTTCAGGATCAGTTCTG**

Ribosomal binding sites are underlined, and binding regions of Gibson oligonucleotides are in bold.

## Data Availability

The data presented in this study are available on request from the corresponding author.

## Supplementary Materials

The following supporting information can be downloaded at: https://www.mdpi.com/article/10.3390/biology11050744/s1, Figure S1: 5-HTP effect on the growth of *C. glutamicum* wild-type, Figure S2: HPLC–MS analysis of 5-FTP production of SER2 pSdS-*trpB* strain cultivated in the presence of 1 mM 5-FI (right) or without (left).
