# Supplementary material for: l-Serine Biosensor-Controlled Fermentative Production of l-Tryptophan Derivatives by Corynebacterium glutamicum
Source: Biology (Basel). 2022 May 13;11(5):744. doi: 10.3390/biology11050744 (PMC9138238; doi:10.3390/biology11050744)
Supplement: Supplementary file 1 [file biology-11-00744-s001.zip › biology-1691637-supplementary.pdf]

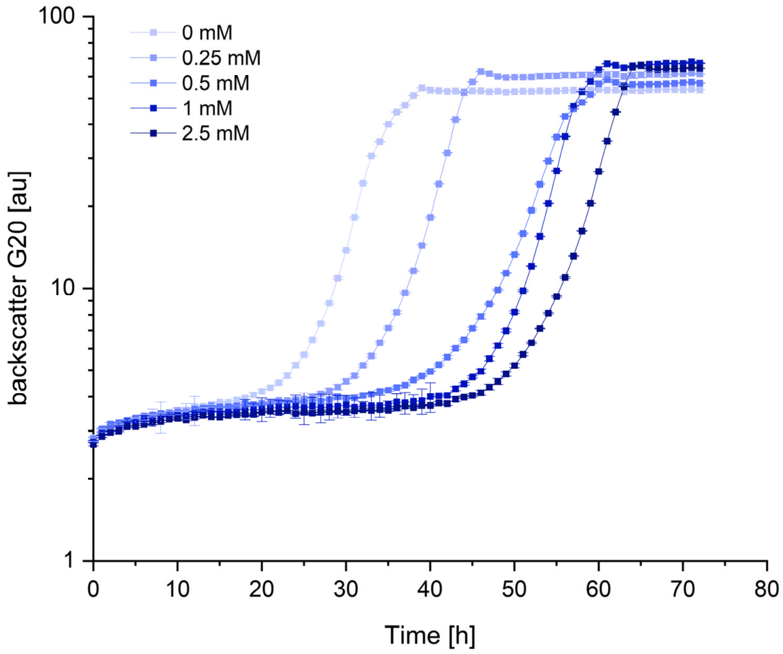

Supplementary Figure S1. 5-HTP effect on the growth of *C. glutamicum* wild-type. WT strain was grown in CGXII minimal medium and cultivated in BioLector micro-cultivation system. 5-HTP was dissolved in MeOH; hence, the MeOH concentration was kept constant at 2 M across all 5-HTP concentrations tested. Means and standard deviations of three replicate cultivations are shown.

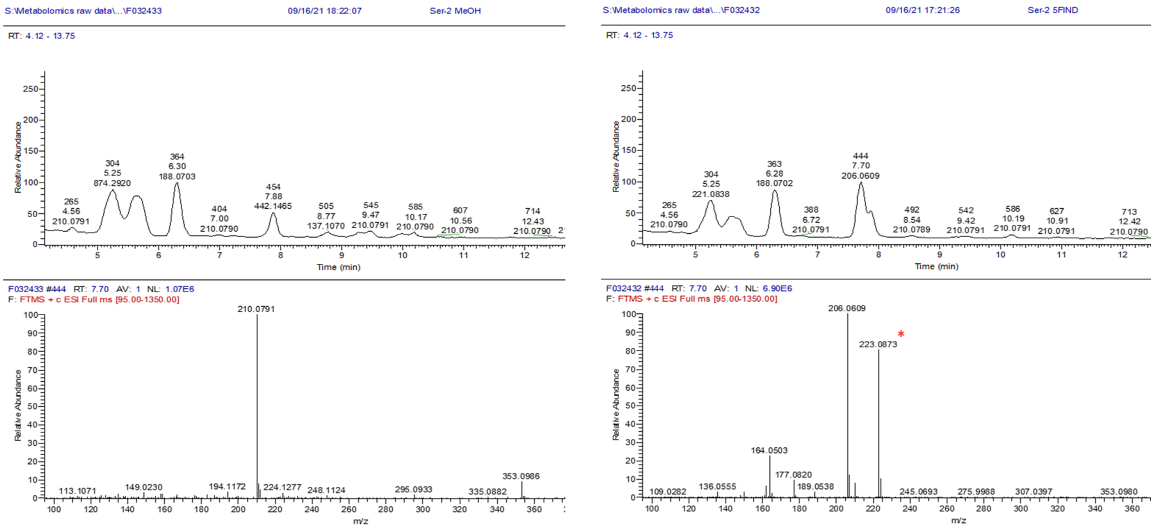

Supplementary Figure S2. HPLC–MS analysis of 5-FTP production of SER2 pSdS-*trpB* strain cultivated in the presence of 1 mM 5-FI (right) or without (left). For the control cultivation without the substrate, 0,124 M MeOH was used instead because 5-FI was dissolved in MeOH.
